# Disability transitions after 30 months in three community-dwelling diagnostic groups in Spain

**DOI:** 10.1371/journal.pone.0077482

**Published:** 2013-10-31

**Authors:** Jesús de Pedro-Cuesta, Pilar García-Sagredo, Enrique Alcalde-Cabero, Angel Alberquilla, Javier Damián, Graciela Bosca, Fernando López-Rodríguez, Monserrat Carmona, María J. de Tena-Dávila, Luis García-Olmos, Carlos H. Salvador

**Affiliations:** 1 Applied Epidemiology, National Centre for Epidemiology and Carlos III Institute of Health, Madrid, Spain; 2 Telemedicine and Health Unit, Carlos III Institute of Health, Madrid, Spain; 3 Multiprofessional Education Unit for Family and Community Care (Centre), Servicio Madrileño de Salud, Madrid, Spain; 4 Bioengineering and Telemedicine Unit, Puerta de Hierro University Teaching Hospital, Madrid, Spain; 5 Multiprofessional Education Unit for Family and Community Care (South-east), Servicio Madrileño de Salud, Madrid, Spain; University of Glasgow, United Kingdom

## Abstract

**Background:**

Little is known about changes in disability over time among community-dwelling patients. Accordingly, this study sought to assess medium-term disability transitions.

**Patients and Methods:**

300 chronic obstructive pulmonary disease (COPD), chronic heart failure and stroke patients living at home in Madrid were selected from general practitioner lists. In 2009, disability was assessed after a mean of 30 months using the World Health Organisation (WHO) *Disability* *Assessment* Schedule *2.0* (WHODAS 2.0). Follow-up was completed using death registries. Losses to follow-up were due to: death, 56; institutionalisation, 9; non-location, 18; and non-participation, 17. Changes in WHODAS 2.0 scores and life status were described and analysed using Cox and multinomial regression. Disability at end of follow-up was imputed for 56 deceased and 44 surviving patients.

**Results:**

Mean disability scores for 200 surviving patients at end of follow-up were similar to baseline scores for the whole group, higher than their own baseline scores, and rose by 16.3% when imputed values were added. The strongest Cox predictors of death were: age over 84 years, adjusted hazard ratios with 95%CI 8.18 (3.06-21.85); severe/complete vs. no/mild disability, 5.18 (0.68-39.48); and stroke compared to COPD, 1.40 (0.67-2.91). Non-participants and institutionalised patients had higher proportions with severe/complete baseline disability. A one-point change in baseline WHODAS 2.0 score predicted independent increases in risk of 12% (8%-15%) for severe/complete disability or death.

**Conclusions:**

A considerably high proportion of community-dwelling patients diagnosed with COPD, CHF and stroke undergo medium-term changes in disability or vital status. The main features of the emerging pattern for this group appear to be as follows: approximately two-thirds of patients continue living at home with moderately reduced functional status; 1/3 die or worsen to severe/complete disability; and 1/10 improve. Baseline disability scores, age and diagnosis are associated with disability and death in the medium term.

## Introduction

Disability assessment in clinical practice has been used to monitor individual patient outcomes. In addition, predicting negative outcomes, such as severe disability or death, can be relevant when it comes to planning care and preventive measures. In this paper, particular attention is given to assessing disability in primary care, whether directly or, in e-health research settings, using Information Communication Technologies (ICTs).

Diverse forms of monitoring appear to provide efficient and effective methods of delivering integrated care in the home health sector [1,2]. Disability, as a component of the routine follow-up of chronically ill elderly persons, has been proposed as a means of improving service provision in cases of frailty [3,4]. In the field of disability monitoring, however, both in traditional care and using ICTs, there is ample room for developing an instrument of choice, examiner profile, indication and adaptation to data-collection procedures. A relevant hindrance to development and application to diagnostic groups is the limited information on expected disability transitions during specific intervals of patients' life course.

The International Classification of Functioning, Disability and Health (ICF) model constitutes an extensive and universally accepted taxonomic classification of disability, which enables clinicians to describe patients' functioning and disability comprehensively and categorise this in a systematic and standardised manner [5,6]. The World Health Organisation (WHO) *Disability Assessment* Schedule *2.0* (WHODAS 2.0), 36 items version (WHODAS-36) is an ICF disability instrument which has successfully shown its usefulness for monitoring patients' outcomes in clinical practice and in clinical trials of treatment effects (for a review see Üstün et al [7]). Versions for self- and lay proxy-informants have been developed.

In 2009, disability was measured using the WHODAS-36 on three groups of Spanish, home-dwelling, primary-care patients who suffered from chronic obstructive pulmonary disease (COPD), chronic heart failure (CHF) and stroke [8]. This instrument was deemed suitable for monitoring the health status of elderly persons living at home [8]. In order to detect negative outcomes in the above cohort, this study focused on assessing disability by the same method and ascertaining mortality across a 30-month follow-up.

## Patients and Methods

### The instrument used

The 36-item version of the WHODAS 2.0 is a disability scale focusing on the ICF Activities and Participation chapters, described in the Table S1. Each item is scored from 0-4; these points are then expressed as a percentage of the maximum theoretical score, and constitute the official disability score of 0-100. This score is, in turn, systematically categorised into no (0%-4%), low (5%-24%), moderate (25%-49%), severe (50%-95%), and extreme or complete (96%-100%) disability. Items addressing occupational status were generically treated, e.g., with work tasks being deemed to be those performed by retired persons actively involved in doing productive unpaid work [9]. The sexuality item was also excluded from the analysis, owing to a high proportion of missing values. The Life Activities domain was assessed in terms of domestic life solely among participants who still performed such activities, i.e., thereby avoiding the need to record the traditionally low performance of men. In line with reported methods [8–10], missing data for items with less than 30% of missing values were replaced by the mean of the remaining domain values, and in cases where 30% or more of values were missing, the domain was left blank. Individuals with more than one blank WHODAS 2.0 domain were excluded from the analysis. The WHODAS-36 has been extensively tested and shown to be an easily administered, robust instrument [7].

### Baseline assessment

A prevalence survey of 26 chronic diseases considered to generate high figures was conducted on the population aged >14 years of the Southern Madrid Region, traditionally a residential area (see *Carmona* et al 2010 for detailed methods and survey CHF outcome [11]). As indicated in a prior report, COPD, CHF and stroke were judged to be diagnostic groups appropriate for a disability study, in view of their higher prevalence and mean individual use of health resources as compared to the above-mentioned remaining 23 chronic conditions [8]. Data, subjected to the application of reported quality criteria, were obtained from the electronic medical records of 129 physicians. Patients were deemed to present with COPD, CHF or stroke where they had an International Classification of Primary Care diagnostic code R79 or R95 for COPD, K77 for CHF, and K90 or K91 for stroke, and had made a medical visit in 2007. This procedure made it possible to identify 3183, 1377 and 2658 patients, who yielded prevalences of 21, 9 and 18 per 1000 population for COPD, CHF and stroke respectively [8]. After verification of life status, a subsample of 1053 patients, balanced in terms of each diagnostic category, was used to obtain residence at home or willingness to participate, with the three convenience samples of patients diagnosed with COPD, 102, CHF, 99, and stroke, 99, constituting the group chosen to undergo disability assessment [8]. During the period April to September 2009, these patients were assessed at home by trained field workers using the WHODAS 36-item version. In essence, the sample corresponded to a community-dwelling population in South Madrid having a low educational and low income level: for a more detailed account of patient selection, the participation rate, details of the WHODAS 2.0 measurements and a description of the sample's educational, economic and social characteristics, readers are referred to the baseline study report [8]. 

The 12 de Octubre Hospital Research Ethics Committee approved the baseline study in February 2009 (report 09/42), and the follow-up study, verbally in September 2011 and formally in January 2011 (by an amendment to the 09/42 report). Participants gave written informed consent to participating in the baseline (W1) assessment and verbal informed consent to being assessed in W2.  Following consultation with the above-mentioned Ethics Committee in September 2011, written consent was not deemed necessary for W2 in view of the fact that both the patients and questions (WHODAS 2.0) used for the purposes of W1 were identical to those used for the purposes of W2.

### Follow-up

In October 2011, after examining The Madrid Regional Death Registry and verifying that 56 (18.7%) out of the 300 patients had died, letters were sent to the remaining 244 patients or their relatives, inviting them to participate in a new evaluation at home and advising them that they would receive a telephone call. Telephone calls were made once or twice from November to December 2011. Eighteen patients (6.0%) were not located and 17 (5.7%) refused to participate. Nine (3%) patients had been institutionalised. One hundred and twenty-nine patients were assessed at home, 14 by proxy. Fifty-eight patients and 13 proxies answered the questionnaire by telephone. In all, 200 patients -77 diagnosed with COPD, 63 with stroke and 60 with CHF- had valid assessment results at baseline (W1) and follow-up (W2). Successful follow-up was achieved for 282 out of the 300 patients, with the above-mentioned 18 patients who proved impossible to locate by mail or telephone being classified as lost to follow-up. 

### Data analysis

Mortality for the different variables was studied using Cox regression models. We obtained hazard ratios (HRs) adjusted for age (attained age was used as the time scale), sex and diagnostic group.

 With regard to disability, total WHODAS scores [10] were calculated and categorised in accordance with the five severity levels described above. In order to study the effect of W1 variables on outcomes, we created a four-category outcome variable encompassing three disability score intervals in W2 (none or low [0-24 points], moderate [25-49 points] and severe/complete [50-100 points]) and deceased during follow-up. In addition, we calculated the probabilities of these outcomes for the same disability categories in W1. Adjusted probabilities were computed with the aid of a multinomial logistic model that included age, sex and diagnostic group.

 In order to assess WHODAS scores on the assumption that all baseline subjects had been measured in W2, we used multiple imputation [12,13] for the 56 subjects who had died and the 44 who had not been completely followed-up. We obtained 10 W2 WHODAS-score imputed data sets using a linear regression model that included age, sex, diagnostic group and W1 WHODAS scores. We then computed pooled mean scores with the 10 imputed data sets, and standard errors by taking the within- and between-imputation components into account. An imputation procedure excluding the 56 deceased patients was also performed.

## Results

**Figure 1:**
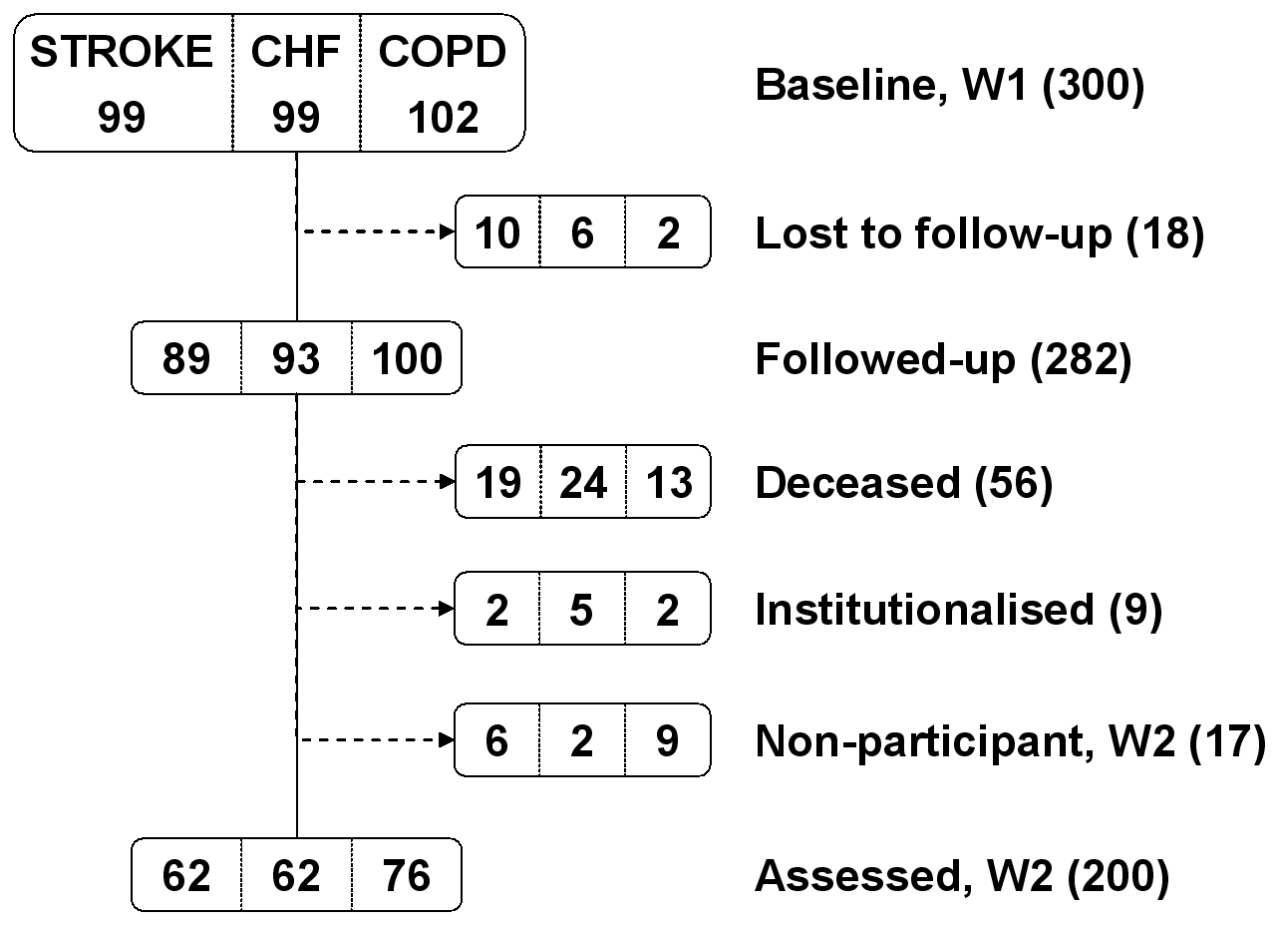
Attrition at follow-up for all patients and diagnostic groups.

 Of the 300 participants at baseline, the above-described 282 were followed up for a median of 2.4 years. In the case of the 18 patients who could not be traced, the reason frequently given by neighbourhood informants to explain this was a change of address, whether the new residence was unknown or known, institutional or non-institutional. Age at baseline of the 282 study participants ranged from 28 to 98 years, with a median of 77 and a mean (SD) of 75 (11.4) years. Figure 1 depicts W1 to W2 attrition, which was 33% overall, and 37% for CHF and stroke, with losses being highest among patients with stroke, 10/99, 10%. The proportion of deceased was highest among those with CHF, 24/99, 24%.

Table 1 shows the characteristics of the 300 patients in W1, and of 200 in W2, both overall and by diagnostic group. Mean W1 WHODAS scores were 33.8 for all patients, 26.4 for COPD, 38.0 for CHF and 37.2 for stroke, and were practically unchanged in W2. Compared to the whole group in W1, the 18 patients lost to follow-up: were an average of 8.8 years younger, with only one subject aged ≥85 years; comprised mainly men, with an F/M ratio of 1/2; had a high stroke frequency (10 subjects) and a higher degree of disability; and registered a mean (SD) WHODAS score of 44.7 (23.5), all of which suggested a profile clearly different from that of baseline participants.

**Table 1:** Descriptive information by diagnostic group and wave.

		COPD		Chronic heart failure		Stroke		Total	
Variable	Category	W1	W2	W1	W2	W1	W2	W1	W2
	All	102 (100) ^a^	76 (100)	99 (100)	62 (100)	99 (100)	62 (100)	300 (100)	200 (100)
Sex	Female	38 (37.3)	29 (38.2)	53 (53.5)	35 (56.5)	46 (46.5)	27 (43.5)	137 (45.7)	91 (45.5)
	Male	64 (62.7)	47 (61.8)	46 (46.5)	27 (43.5)	53 (53.5)	35 (56.5)	163 (54.3)	109 (54.5)
Age (years)	<75	23 (22.5)	17 (22.4)	9 ( 9.1)	6 ( 9.7)	17 (17.2)	9 (14.5)	49 (16.3)	32 (16.0)
	75-84	24 (23.5)	14 (18.4)	16 (16.2)	10 (16.1)	26 (26.3)	18 (29.0)	66 (22.0)	42 (21.0)
	≥85	55 (53.9)	45 (59.2)	74 (74.7)	46 (74.2)	56 (56.6)	35 (56.5)	185 (61.7)	126 (63.0)
Marital status	Without spouse/partner	39 (38.2)	29 (38.2)	49 (49.5)	25 (40.3)	35 (35.4)	26 (41.9)	123 (41.0)	80 (40.0)
	With spouse/partner	63 (61.8)	47 (61.8)	50 (50.5)	37 (59.7)	64 (64.6)	36 (58.1)	177 (59.0)	120 (60.0)
Occupational status	Active	18 (17.6)	15 (19.7)	12 (12.1)	14 (22.6)	14 (14.1)	11 (17.7)	44 (14.7)	40 (20.0)
	Retired	78 (76.5)	60 (78.9)	82 (82.8)	47 (75.8)	81 (81.8)	48 (77.4)	241 (80.3)	155 (77.5)
	Other	6 ( 5.9)	1 ( 1.3)	5 ( 5.1)	1 ( 1.6)	4 ( 4.0)	3 ( 4.8)	15 ( 5.0)	5 ( 2.5)
Baseline living/residential situation	Independent-no services	67 (65.7)	53 (69.7)	40 (40.8)	33 (53.2)	43 (44.3)	29 (48.3)	150 (50.5)	115 (58.1)
	Family support	20 (19.6)	14 (18.4)	34 (34.7)	19 (30.6)	38 (39.2)	25 (41.7)	92 (31.0)	58 (29.3)
	Professional help	15 (14.7)	9 (11.8)	24 (24.5)	10 (16.1)	16 (16.5)	6 (10.0)	55 (18.5)	25 (12.6)
Educational level	Less than primary	16 (15.7)	7 ( 9.2)	10 (10.5)	8 (12.9)	9 ( 9.1)	2 ( 3.2)	35 (11.8)	17 ( 8.5)
	Primary	43 (42.2)	33 (43.4)	43 (45.3)	31 (50.0)	42 (42.4)	37 (59.7)	128 (43.2)	101 (50.5)
	Secondary or higher	43 (42.2)	36 (47.4)	42 (44.2)	23 (37.1)	48 (48.5)	23 (37.1)	133 (44.9)	82 (41.0)
Disability level	No/Mild	56 (54.9)	47 (61.8)	32 (32.3)	20 (32.3)	41 (41.4)	24 (38.7)	129 (43.0)	91 (45.5)
	Moderate	31 (30.4)	14 (18.4)	38 (38.4)	27 (43.5)	25 (25.3)	23 (37.1)	94 (31.3)	64 (32.0)
	Severe/complete	15 (14.7)	15 (19.7)	29 (29.3)	15 (24.2)	33 (33.3)	15 (24.2)	77 (25.7)	45 (22.5)
Disability score, mean (SD)		26.4 (20.8)	26.7 (20.9)	38.0 (23.2)	36.2 (19.2)	37.2 (27)	35.7 (25.6)	33.8 (24.3)	32.5 (22.3)

Abbreviations: COPD: chronic obstructive pulmonary disease. W1, W2: Waves 1 and 2.

a Absolute numbers (percentages).

**Figure 2:**
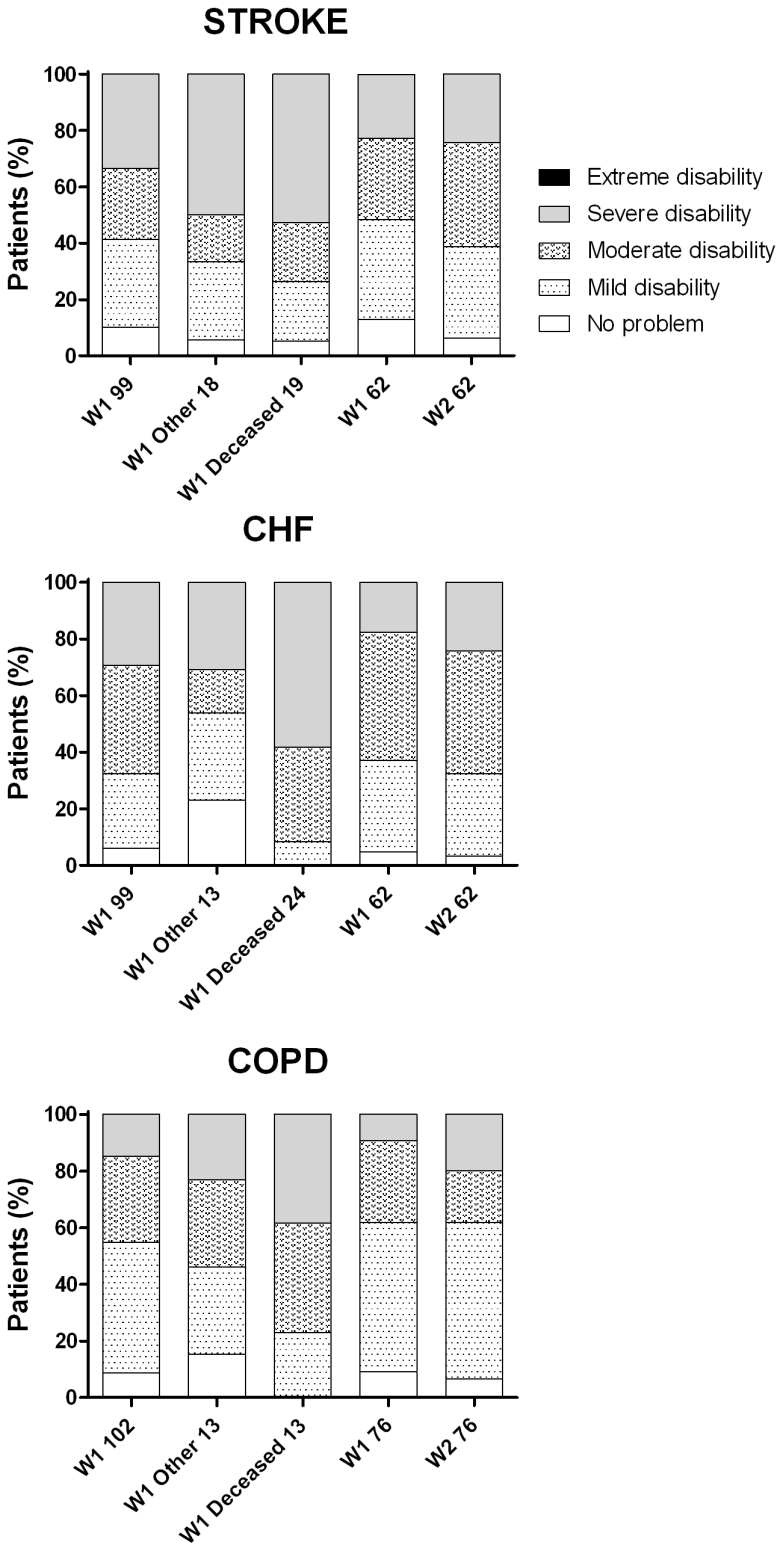
Prevalence of disability categories in three diagnostic groups and several subgroups: all patients at baseline (left); other=non-participants and institutionalised (centre left); deceased during follow-up (centre); and followed-up community-dwelling residents at baseline (centre right) and end of follow-up (right).

 A detailed breakdown of the study population by diagnostic group is depicted in Figure 2, in which disability categories from direct assessments are shown for each diagnostic group and subgroup: firstly in W1, for all patients, for those who were followed-up, for those who died and for the subgroup denoted as "other", including those lost to follow-up and non-participants; and secondly in W2, for all those who were assessed. In W1, the proportions of severely disabled among deceased and "other" (lost and non-participant) patients were systematically the highest. For those assessed in W1 and W2, the proportions of severely disabled remained unchanged for stroke, while a worsening trend was suggested for CHF and COPD. The corresponding mean W1 scores of 30.7, 32.3 and 22.6 worsened to 35.7, 36.2 and 26.7 respectively. In brief, in W1, patients who were not assessed in W2 and those who subsequently died were more disabled than those who were assessed after follow-up; all diagnostic groups worsened across follow-up.

 The mortality rate for the population aged ≥60 years was 99.0 per 1000 person-years (fifty-six deaths during 565 person-year follow-up); the cumulative incidence of death was 20% (56/282) (95%CI: 15-25%). Adjusted hazard ratios (HRs) for death are shown in Table 2. Higher HRs were seen for subjects with ages ≥85 years at baseline (HR= 8.18), severe/complete disability (HR= 5.18) and need of professional help (HR= 3.24). A breakdown by diagnosis showed that the highest mortality rate was attributable to stroke, followed by CHF and COPD. No association was seen with resource utilisation level [14].

**Table 2:** Mortality hazard ratios for demographic and disability variables.

Variable	Category	HR ^a^ (95% CI)
Sex	Female	1
	Male	0.71 (0.40-1.26)
Age (years)	<75	1
	75-84	4.79 (1.84-12.45)
	≥85	8.18 (3.06-21.85)
Marital status	With no spouse/partner	1
	With spouse/partner	0.99 (0.52-1.89)
Occupational status	Active	1
	Retired	2.73 (0.63-11.93)
	Other ^b^	6.10 (0.97-38.51)
Living/residential situation	Independent-no services	1
	Family support	1.78 (0.83-3.85)
	Professional help	3.24 (1.48-7.09)
Educational level	Less than primary	1
	Primary	0.54 (0.23-1.28)
	Secondary and higher	0.86 (0.36-2.02)
Diagnostic group	COPD	1
	Chronic heart failure	1.19 (0.58-2.44)
	Stroke	1.40 (0.67-2.91)
Disability level	No problem	1
	Mild disability	1.52 (0.19-12.32)
	Moderate disability	3.22 (0.42-24.92)
	Severe/complete disability	5.18 (0.68-39.48)
Resource utilisation bands [14]	Moderate/low cost	1
	High cost	1.33 (0.68-2.70)

a Hazard ratio, adjusted for age, sex and diagnostic group

b On temporary leave, pre-retirement

Table 3 shows the probabilities of a patient classified in any given disability category in W1 being in the same or a different disability category or having died in W2; based on raw data, the probability was highest for the transition from severe disability to death, 47.5% (35.4%-60.0%). Of the 300 patients: 112, 37.3%, worsened to severe/complete disability or death; 25, 8.3%, worsened to moderate disability; and 32, 10.7%, improved to moderate or lower disability. The remaining 131, 43.7%, remained at the same disability level. Improvement from severe/complete to no/mild disability scores was unlikely. Adjustment for age, sex, and diagnosis did not substantially modify the results.

**Table 3:** Probability of follow-up outcome by baseline disability level. Estimates obtained from multinomial logistic regression models.

			Outcome Probability, % (95% CI)			
	Baseline disability level	No. of patients	No/mild	Moderate	Severe/complete	Death
Crude^a^	No/mild	110	64.5 (55.2-72.9)	19.1 (12.8-27.5)	7.3 (3.7-13.9)	9.1 (5.0-16.1)
	Moderate	85	22.4 (14.7-32.4)	41.2 (31.2-51.9)	16.5 (10.0-25.9)	20.0 (12.8-29.8)
	Severe/complete	61	1.6 (0.2-10.7)	13.1 (6.7-24.1)	37.7 (26.5-50.4)	47.5 (35.4-60.0)
Adjusted^b^	No/mild	110	61.8 (51.1-71.5)	22.6 (15.1-32.6)	7.0 (3.4-14.1)	8.5 ( 4.3-16.0)
	Moderate	85	19.7 (11.9-30.7)	44.1 (32.9-55.9)	17.5 (10.3-28.1)	18.8 (11.3-29.5)
	Severe/complete	61	1.9 (0.2-13.5)	15.2 (7.3-28.8)	41.9 (27.9-57.5)	41.0 (27.0-56.6)

a Correspond to overall values in Figure 4

b Adjusted for age, sex and diagnosis.

**Figure 3:**
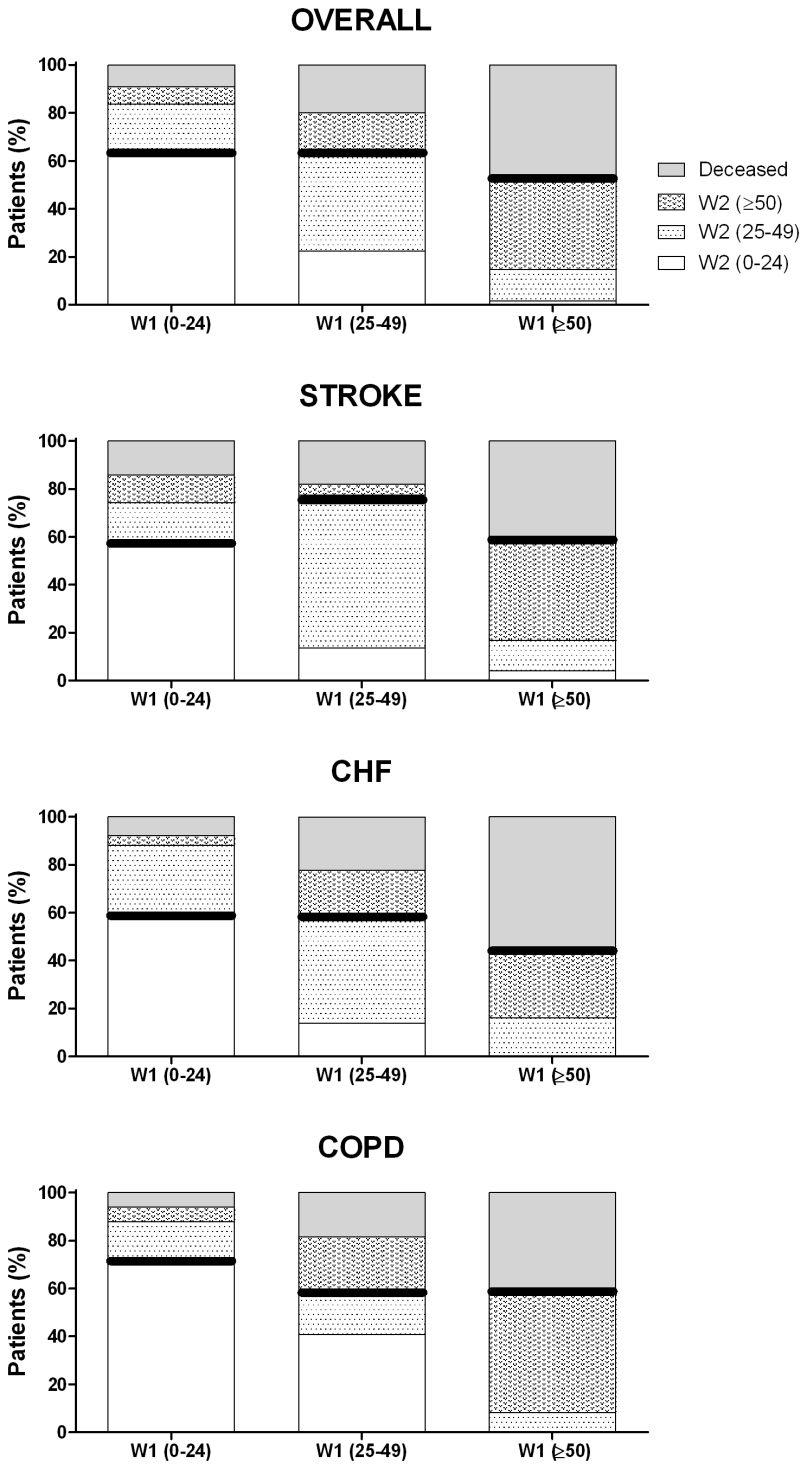
Distribution of outcomes (disability levels and death) at end of follow-up for each of three baseline disability levels, overall and by diagnostic group. Fragments above solid, thick bands indicate worsening with respect to baseline values.

A post-follow-up view of the above-mentioned three disability categories (none/mild, moderate, severe/complete), overall and by diagnostic subgroup, is graphically depicted in Figure 3. Worsening (transition to higher disability or death) was 39% overall, with the corresponding figures by diagnostic group being 37% for stroke, 45% for CHF and 34% for COPD. In the case of diagnostic and disability sub-groups, worsening was approximately 1/3 for disability sub-categories, proving lowest for COPD with no/mild disability and stroke with moderate disability, both of which displayed high proportions with the same disability levels in W1 and W2. The vast majority of patients who were severely/completely disabled in W1, score ≥50, remained severely disabled in W2 or had died, both overall and by group, with the highest mortality seen for CHF.

 Predictors for outcome categories of disability and death were analysed for 256 patients, using multinomial logistic regression models (Table 4) where relative risk ratios (RRRs) for each outcome are shown, taking no/mild disability as the reference outcome category. Age and W1 disability appeared to be strong, systematic predictors of negative outcomes (disability and death). The relative risk ratio per year of age, was highest for severe/complete disability (RRR= 1.13 (1.07-1.19)) and death (RRR=1.15 (1.09-1.2)). Similarly, high increases in risk per disability point score were seen for severe/complete disability or death (RRR= 1.12 (1.08-1.12) in both). Available family support and married status were associated with severe/complete disability in W2 (RRR=10.72 (2.99-38.45) and RRR=4.56 (1.39-14.93) respectively).

**Table 4:** Outcome relative risk ratios from multinomial models.

Variable	Category	Moderate disability	Severe/complete disability	Death
Sex	Male	0.51 (0.24-1.09)	1.09 (0.42-2.86)	0.94 (0.37-2.41)
Age, per 1 year		1.05 (1.01-1.08)	1.13 (1.07-1.19)	1.15 (1.09-1.22)
Marital status	With spouse/partner	1.08 (0.47-2.46)	4.56 (1.39-14.93)	1.51 (0.50-4.51)
Occupational status,	Retired/ other	1.25 (0.4-3.88)	0.35 (0.09-1.46)	2.04 (0.33-12.52)
Living/residential situation	Independent-no services	1	1	1
	Family support	1.71 (0.64-4.57)	10.72 (2.99-38.45)	2.94 (0.88-9.77)
	Professional help	0.80 (0.20-3.24)	1.88 (0.37-9.46)	1.61 (0.37-7.04)
Educational level	Less than primary	1	1	1
	Primary	0.47 (0.13-1.64)	0.65 (0.14-2.95)	0.49 (0.11-2.22)
	Secondary or higher	0.18 (0.05-0.67)	0.76 (0.16-3.55)	0.82 (0.18-3.72)
Diagnostic group	COPD	1	1	1
	Chronic heart failure	2.43 (0.96-6.12)	0.71 (0.23-2.22)	1.19 (0.39-3.68)
	Stroke	3.48 (1.39-8.68)	1.62 (0.52-5.03)	2.28 (0.72-7.16)
W1 Disability Score, per 1-point		1.07 (1.04-1.10)	1.12 (1.08-1.15)	1.12 (1.08-1.15)

Abbreviations: COPD: chronic obstructive pulmonary disease.Adjusted for age, sex, diagnostic group and baseline WHODAS-2 score

No/mild disability taken as reference outcome category

**Figure 4:**
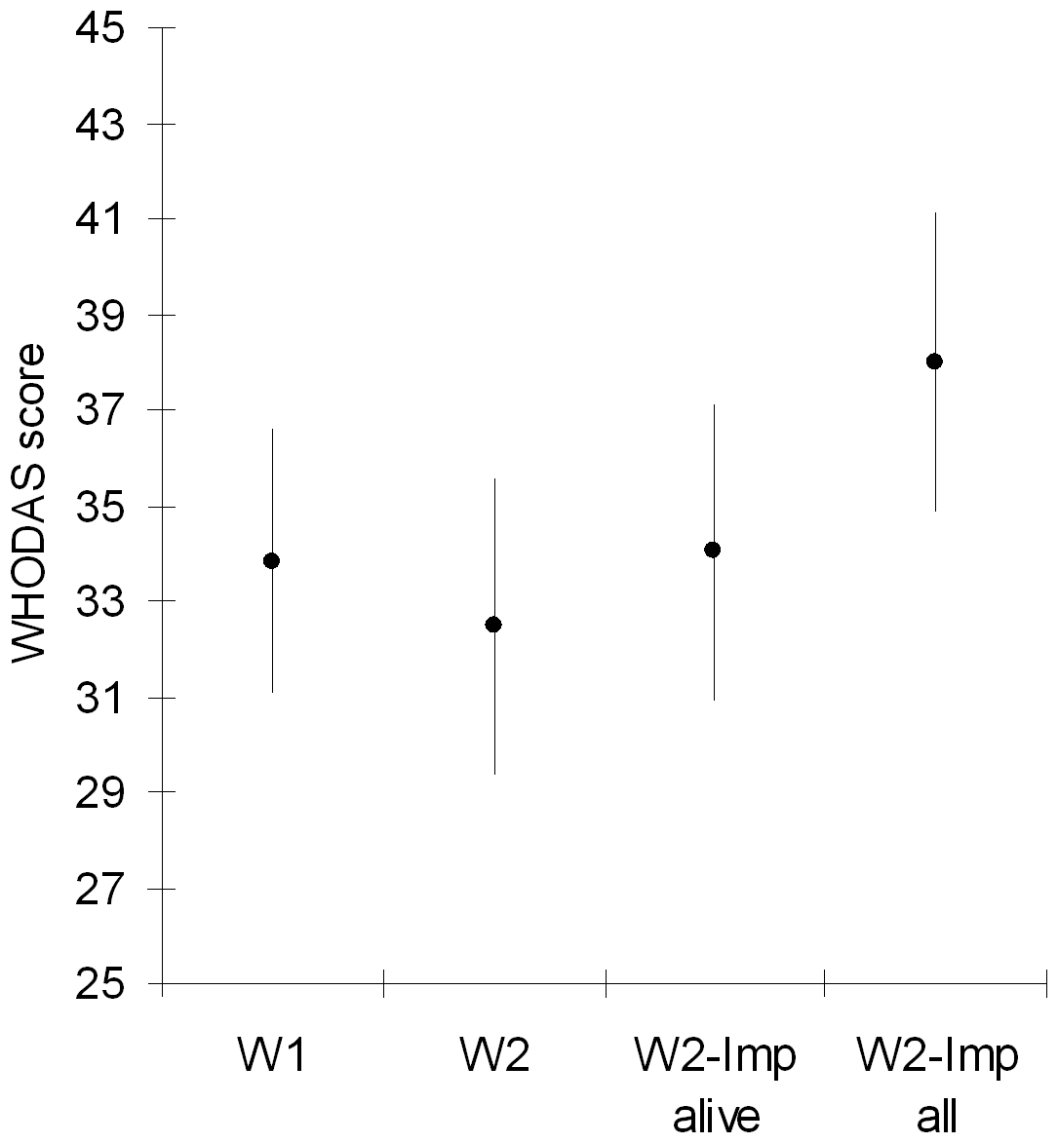
Mean and 95%CI of WHODAS disability scores for: all patients at baseline (W1, left); survivors at end of follow-up (W2, centre-left); survivors at end of follow-up in W2, with imputed values for subjects alive but not-assessed in W2 (centre-right); and, survivors at end of follow-up in W2, with imputed values for all not assessed in W2, including deceased (right).

Figure 4 shows mean disability scores with their 95%CIs for: (a) 300 patients in W1, 33.8; (b) 200 patients assessed in W2, 32.5; (c) 200 patients assessed in W2, with imputed values for 41 surviving patients, 34.0; and, (d) 200 survivors plus imputed values for 96 patients who were dead or alive, 37.8. While minor differences were seen between comparisons using only direct assessments in W1 and W2, and assessed plus imputed values for surviving patients, the fourth group, in which imputed values for deceased patients were included, displayed the highest values, i.e., 11.8% higher than those assessed in W1 and 16.3% higher than those assessed in W2.

## Discussion

The results of this study describe the main features of disability across time among persons who were living at home in a Southern European suburban environment and were chronically ill with one of three somatic disorders. Our follow-up disclosed: (a) a considerable impact of death on sample attrition; (b) a modest increase in disability among survivors; and, (c) similar main determinants of death or worsened disability, i.e., age, baseline disability and diagnosis. Limitations on interpreting results were determined by losses during follow-up, instability linked to sample size, and a lack of control based on relevant variables such as cognitive status or co-morbidity. Since death registration among residents of the Madrid Region is acknowledged to be of high quality, any potential selection bias in losses to follow-up would mainly affect disability. External validity is limited by choice of diagnosis and recruitment. The good agreement between self- and proxy-reported current health problems of elderly people [15] and the low proportion of proxy responses, 14.6% overall, suggest a low underlying bias. 

Mortality was high in this group. Compared to the general population (based on age- and sex-specific mortality rates obtained through official statistics for the Madrid Region, 2010), mortality was more than double, with a standardised mortality ratio (SMR) of 2.11 (95%CI, 1.56-2.67). However, the corresponding figures stratified by sex revealed a clear difference, with SMR=1.37 (95%CI, 0.82-1.92) for men and SMR=2.94 (95%CI, 1.92-3.97) for women. Although the overall high mortality of the study sample was expected, the differential pattern by gender was unforeseen because here in Spain registered age-adjusted vascular-disease and all-cause mortality is lower among women than among men [16].

As seen from multinomial analysis, baseline age and disability are clear predictors of severe disability and death. Such associations fit the well-known pattern of transitional disability, i.e. generally worsening with proximity to death [17]. Since death removes persons from the population at risk of disability, and severe disability in particular, a competing risks scenario is suggested. Nevertheless, its relevance for use of specific analytical methods [18] in disability follow-up studies is beyond the scope of this paper.

Several associations may be related to community living. Living at home with professional help (mainly aid with domestic tasks) is associated with death, and living with family support is strongly associated with severe disability, taking independent living as reference. The magnitude of the association with professional help decreased when adjusting for baseline disability (see Table 4), something that suggests confounding and, in turn, that other factors would determine the link between professional help and disability in W2. In all likelihood, such associations could in part be explained by resource availability, a feature that makes it possible for some severely disabled persons to live at home. 

The finding of high disability in W2 when imputed values for deceased patients were included, is consistent with the predictive value of disability for death and may suggest that transition to death was framed in a scenario where, in addition to high disability scores, a worsening in disability was expected. The difference in mean disability scores between patients assessed in W1 and assessed and imputed values for patients alive in W2 may suggest disability-related loss or non-participation. Lower disability among younger cohorts has been described in Spanish elderly [19,20] and other populations. An emerging effect of mortality removing older birth cohorts in our results is unlikely, however.

The moderate disability increase registered by each diagnostic group between W1 and W2 appears to be in line with the natural history of COPD and CHF as known, progressive disorders. For stroke, a disorder generally followed by at least partial recovery, a worsening in disability might not have been anticipated if patients with recent stroke had been included in sample. Recruitment in 2007 implies, not only that patients were assessed in W1 and W2, several years after the episode determining stroke diagnosis, but also that recovery had already taken place. The fact that patients followed up across the 12 month-5 year interval after the stroke episode in Stockholm showed a significant decrease in the proportion of independent living according to the Katz Extended ADL Index [21], suggests that our findings of increased disability long after stroke are consistent with reported functional loss in the life course of elderly stroke patients.

Relationships between diagnoses, disease course and disability in W1 and W2 in our sample can be complex. A straightforward interpretation of the associations, whereby all disability is attributed to the effect of the specific diagnosis, is probably misleading, despite the fact that CHF, COPD and stroke may determine disability, for the simple reason that disability determined by other health conditions cannot be ruled out. For instance, a random sample of Finnish subjects aged 65-74 years showed that, after adjustment for age and co-morbidity, cerebrovascular diseases in men and myocardial infarction, heart failure and cerebrovascular diseases in women were significantly associated with disability [22]. Nevertheless, only part of the disability -33% in men and 24% in women- was attributable to cardiovascular disease, excluding hypertension alone. This would suggest that, in our sample, CHF-, COPD- and stroke-related disability were pooled with disability of other aetiologies, and age-related co-morbidity in particular. Moreover, since in adults at risk of stroke, disproportionate limitations in activities of daily living, attributed to stroke risk factors such as diabetes, emerge well before stroke onset [23], W1 and W2 disability should not be entirely attributed to the effects of acute stroke.

Different disability trajectories in COPD, CHF and stroke might be attributable to differential co-morbidity, a feature reflected by their inclusion as components of different reported multimorbidity patterns. Using factor analysis on large prevalent samples of primary care diagnoses of chronic conditions among the elderly, two groups in Germany and Spain identified three and four similar patterns respectively [24,25]. In both studies, stroke constitutes a component of the factor denoted as “neuropsychiatric disorders” or “psychogeriatric”, where dementia and other highly disabling degenerative diseases are present and COPD remains a factor-unrelated condition. In contrast, CHF is included, particularly among elderly women, in two factors, namely, “psychogeriatric”, as mentioned above, and “cardio/metabolic”. It could be speculated that a fraction of the excess disability and mortality seen for CHF and stroke as compared to COPD, may be due to differential co-morbidity, and that co-morbidity may serve to explain similarities better than it does to explain differences in transitions between stroke and CHF. 

An additional difficulty when it comes to interpreting associations between diagnoses representing binary categories of health conditions which are partly treatable and thus represent different severity levels, is that the disease-disability association can be bidirectional, inasmuch as disability may worsen recovery or disease course. This is well illustrated by the many (often nursing) reports of studies on CHF patients, aimed at assessing the impact on CHF course of limitations in self-care, attributed, among other things, to personality, depression or cognitive decline [26–28]. In contrast, adequate self-care was linked to an improvement in health status, a decrease in the number and duration of hospitalisations, and a decline in the levels of stress biomarkers and intrathoracic impedance [29]. Hence, if optimising self-care might, in theory, help improve disease course and prognosis, one could hypothesise that a negative disease course due to limitations in self-care might bring about a worsening to higher disability or death, at least in the case of CHF patients. Unfortunately, the effect of interventions to enhance self-management support for patients with COPD and other chronic conditions is still being debated [30].

A tantalisingly similar interpretation would be that the sex differential in disability may be the factor determining the sex differential observed in mortality. It is well known that among elderly women, prevalence of disability is higher than among elderly men, and this trait has also been described by WHODAS 36-item measurements in Spanish populations aged ≥75 years [31]. The fact that global baseline disability in our sample, particularly for stroke patients, was higher among women (odds ratio 2.80 95%CI 1.12-6.97) [8] might support such an interpretation. One could speculate that co-morbidity, rather than limitations in self-care alone, generating the higher prevalence of disability among women might also contribute to the sex differential in the mortality pattern observed.

 In this study attention has focused on global disability scores, neglecting the picture for specific domains such as *Getting around* or *Self-care*. A domain-specific approach might be more sensitive to specific disability transitions, since domain-specific disability, even at a high level, can be concealed by dilution in global scores. Individual monitoring targets including disability, may differ for diagnoses and for elderly presenting with multiple diagnoses, and may therefore have to be individually designed. Abete et al suggest that self-care management, caregiver training and multiprofessional teams represent the critical point for treatment of elderly CHF patients and that follow-up of elderly CHF patients is extremely important [32]. Similar approaches have been suggested for home-based rehabilitation of stroke patients, where combined monitoring of rehabilitation, anticoagulation and other techniques have been tested [33,34]. In future, appropriate disability monitoring design, including indication, may go towards maximising the still limited evidence supporting the contention that ICT-based monitoring performs better than does traditional care in reducing both the decline in frailty states and death [35].

## Conclusions

To sum up, this study shows that disability and death are associated with baseline disability scores, age and diagnosis. At 30 months, two out of three COPD, CHF and stroke patients continued living at home with reduced functional status, one in three had died or worsened to severe/complete disability, and one in ten had improved to lower disability. Transitions by disability domain may differ from results for global disability and warrant specific approaches. 

## Supporting Information

Table S1 **World Health Organisation Disability Assessment Schedule 2.0, 36 items (WHODAS-2 36-item) over six domains, with the corresponding International Classification of Functioning, Disability and Health (ICF) codes.**
(DOC)
