# Correction: Disability transitions after 30 months in three community-dwelling diagnostic groups in Spain

**DOI:** 10.1371/annotation/1662bc65-ded5-4918-ad36-16e7e3fc65a4

**Published:** 2013-12-11

**Authors:** Jesús de Pedro-Cuesta, Pilar García-Sagredo, Enrique Alcalde-Cabero, Angel Alberquilla, Javier Damián, Graciela Bosca, Fernando López-Rodríguez, Montserrat Carmona, María J. de Tena-Dávila, Luis García-Olmos, Carlos H. Salvador

The eighth author's name was spelled incorrectly. The correct name is: Montserrat Carmona.

The title of Reference 16 is incorrect. The corrected Reference 16 is:

16. Instituto de Salud Carlos III website. (2010) [Mortalidad por Capítulo, Causa y Sexo (2010)]. http://www.isciii.es/ISCIII/es/contenidos/fd-servicios-cientifico-tecnicos/fd-vigilancias-alertas/M_10_Cap_cau.xls. Accesssed 17 September 2013.

In the footnote of Table 3, the text for a. references Figure 4 erroneously. The correct text should read: "a. Correspond to overall values in Figure 3." 

