# Supplementary material for: Disability transitions after 30 months in three community-dwelling diagnostic groups in Spain
Source: PLoS One. 2013 Oct 31;8(10):e77482. doi: 10.1371/journal.pone.0077482 (PMC3879317; doi:10.1371/journal.pone.0077482)
Supplement: Table S1 — World Health Organisation Disability Assessment Schedule 2.0, 36 items (WHODAS-2 36-item) over six domains, with the corresponding International Classification of Functioning, Disability and Health (ICF) codes. (DOC) [file pone.0077482.s001.doc]

Supplementary Table S1. World Health Organisation Disability Assessment Schedule 2.0, 36 items (WHODAS-2 36-item) over six domains, with the corresponding International Classification of Functioning, Disability and Health (ICF) codes.^a,b^

| Domain and item | Domain question | ICF code |
| --- | --- | --- |
| **D1: Understanding and communicating** | **In the last 30 days, how much difficulty did you have in:** |  |
| D1.1 | Concentrating on doing something for 10 minutes | d160 Focusing attention; b140 Attention functions; d110-d129 Purposeful sensory experiences |
| D1.2 | Remembering to do important things | b144 Memory functions |
| D1.3 | Analysing and finding solutions to problems in day-to-day life | d175 Solving problems; d130-d159 Basic learning |
| D1.4 | Learning a new task, for example, learning how to get to a new place | d1551 Acquiring complex skills |
| D1.5 | Generally understanding what people say | d310 Communicating with - receiving - spoken messages |
| D1.6 | Starting and maintaining a conversation | d3500 Starting a conversation; d3501 Sustaining a conversation |
| **D2: Getting around** | **In the last 30 days, how much difficulty did you have in:** |  |
| D2.1 | Standing for long periods such as 30 minutes | d4154 Maintaining a standing position |
| D2.2 | Standing up from sitting down | d4104 Standing |
| D2.3 | Moving around inside your home | d4600 Moving around within the home |
| D2.4 | Getting out of your home | d4602 Moving around outside the home and other buildings |
| D2.5 | Walking a long distance such as a kilometre (or equivalent) | d4501 Walking long distances |
| **D3: Self-care** | **In the last 30 days, how much difficulty did you have in:** |  |
| D3.1 | Washing your whole body | d5101 Washing whole body |
| D3.2 | Getting dressed | d540 Dressing |
| D3.3 | Eating | d550 Eating |
| D3.4 | Staying by yourself for a few days | d510-d650 Combination of multiple self-care and domestic life tasks |
| **D4: Getting along** | **In the last 30 days, how much difficulty did you have in** |  |
| D4.1 | Dealing with people you do not know | d730 Relating with strangers |
| D4.2 | Maintaining a friendship | d7500 Informal relationships with friends |
| D4.3 | Getting along with people who are close to you | d760 Family relationships; d770 Intimate relationships; d750 Informal social relationships |
| D4.4 | Making new friends | d7500 Informal relationships with friends; d7200 Forming relationships |
| D4.5 | Sexual activities | d7702 Sexual relationships |
| **D5: Life activities** | **In the last 30 days, how much difficulty did you have in:** |  |
| D5.1 | Taking care of your household responsibilities | d6 Domestic life |
| D5.2 | Doing most important household tasks well | d640 Doing housework; d210 Undertaking a single task; d220 Undertaking multiple tasks |
| D5.3 | Getting all the household work done that you needed to do | d640 Doing housework; d210 Undertaking a single task; d220 Undertaking multiple tasks |
| D5.4 | Getting your household work done as quickly as needed | d640 Doing housework; d210 Undertaking a single task; d220 Undertaking multiple tasks |
| D5.5 | Your day-to-day work/school | d850 Remunerative employment; d830 Higher education; d825 Vocational training; d820 School education |
| D5.6 | Doing your most important work/school tasks well | d850 Remunerative employment; d830 Higher education; d825 Vocational training; d820 School education; d210 Undertaking a single task; d220 Undertaking multiple tasks |
| D5.7 | Getting done all the work that you needed to do | d850 Remunerative employment; d830 Higher education; d825 Vocational training; d820 School education; d210 Undertaking a single task; d220 Undertaking multiple tasks |
| D5.8 | Getting your work done as quickly as needed | d850 Remunerative employment; d830 Higher education; d825 Vocational training; d820 School education; d210 Undertaking a single task; d220 Undertaking multiple tasks |
| **D6: Participation in society** | **How much of a problem do you have:** |  |
| D6.1 | Joining in community activities | d910 Community life |
| D6.2 | Because of barriers or hindrances in the world | d9 Community, social and civic life |
| D6.3 | Living with dignity | d940 Human rights |
| D6.4 | From time spent on health condition | Not applicable (impact question) |
| D6.5 | Feeling emotionally affected | b152 Emotional functions |
| D6.6 | Because health is a drain on your financial resources | d8700 Personal economic resources |
| D6.7 | With your family facing difficulties due to your health | Not applicable (impact question) |
| D6.8 | Doing things for relaxation or pleasure by yourself | d920 Recreation and leisure |

^a^The WHODAS 2.0 also includes two preliminary sections which enquire about demographic variables and general health. These sections are to be used if the WHODAS 2.0 is used alone but may be dropped or modified if WHODAS 2.0 is used in conjunction with other instruments that already collect such information. A final optional section enquires about the attributes and impact of identified problems.

^b^Modified from Üstün *et al* 2010 [7].
